# The first line of defence: insights into mechanisms and relevance of phagocytosis in epithelial cells

**DOI:** 10.1007/s00281-018-0701-1

**Published:** 2018-09-04

**Authors:** Juliane Günther, Hans-Martin Seyfert

**Affiliations:** Institute for Genome Biology, Leibniz Institute for Farm Animal Biology, 18196 Dummerstorf, Germany

**Keywords:** Epithelial cells, Phagocytosis, Pathogen recognition, Commensals, Tolerance, Dead cell clearance

## Abstract

Epithelial tissues cover most of the external and internal surfaces of the body and its organs. Inevitably, these tissues serve as first line of defence against inorganic, organic, and microbial intruders. Epithelial cells are the main cell type of these tissues. Besides their function as cellular barrier, there is growing evidence that epithelial cells are of particular relevance as initial sensors of danger and also as executers of adequate defence responses. These cells feature various essential functions to maintain tissue integrity in health and disease. In this review, we survey some of the different innate immune functions of epithelial cells in mucosal tissues being constantly exposed to a plethora of harmless contaminants but also of pathogens. We discuss how epithelial cells avoid inadequate immune responses in such conditions. In particular, we will focus on the diverse types and mechanisms of phagocytosis used by epithelial cells to not only maintain homeostasis but to also harness the host response against invading pathogens.

## Introduction: immunocompetence of epithelial cells

Epithelia cover the external surfaces of the body, line body cavities, and the tubes connecting them with the environment. Stratified epithelia build a barrier to the environment (skin). Squamous epithelia line organs and contribute to the building of organs with highly specialised functions. These include absorption (e.g. lung, gut) and secretion (e.g. mammary gland, kidney, and stomach) as well as entry and exit of material (e.g. trachea, oral/nasal cavity, ureter, and vagina). Depending on their position, epithelia intensively communicate with their external surrounding. They constitute the first line of defence against invading pathogens. This relates in particular—but not exclusively—to the efferent and supplying hollow organs such as trachea or ducts of the mammary gland. Epithelial cells are by far the most abundant cell type in these tissues. In recent years, it has become more and more clear that they contribute crucially to initiating and governing the initial steps of the immune response [1–6]. Not only do they build a physical barrier against harmful substances and pathogens, but they also exert manifold sentinel functions in perceiving pathogens and orchestrating the defence against them. In addition, they sustain tissue homeostasis by modulating the composition of their surrounding milieu and the responsiveness of resident professional immune cells.

## Sensor functions of epithelial cells

“Be aware of the danger—but recognize the opportunity” (J.F. Kennedy)

Rapid recognition of pathogens and other potentially dangerous incidents is of critical importance for a benign outcome of diseases. Swift recognition facilitates the timely initiation of an adequate response to eliminating the threatening situations. Amongst others, pathogen recognition is known to facilitate phagocytosis by binding and engulfing of the pathogen. Furthermore, signalling pathways activated by attacking pathogen or danger recognition are linked to the lysosomal degradation pathways. However, epithelia not exclusively challenged with dangerous insults, but rather are day-by-day confronted with harmless microbes and innocuous contaminants. This makes it necessary to tightly control these sensory pathways to avoid immunopathology.

### Pattern recognition receptors: sensing enemies and danger

Infections are perceived by cells trough specific pathogen recognition receptors (PRRs) detecting microbial compounds (pathogen-associated molecular patterns, PAMPs). Prototypical examples of those immune stimulatory microbial compounds are components of the pathogen surface like lipopolysaccharide (LPS), peptidoglycan, and flagellin but also bacterial and viral nucleic acid. Other harmful situations may be recognised through endogenous danger-associated signals (danger-associated molecular patterns, DAMPs) released from stressed or damaged cells [7]. These can be proteins such as the chromatin-associated high-mobility group box 1 (HMGB1) and S100 proteins or non-proteins such as ATP and host DNA or RNA that are normally hidden inside the cell. Epithelial cells perceive various PAMPs and DAMPs through diverse sets of sensors including Toll-like receptors (TLRs), nucleotide-binding oligomerization domain-containing proteins (NODs), Dectin-1, Galectins, and retinoic acid-inducible gene 1 (RIG-I) [8, 9].

TLRs are membrane-bound PAMP sensors expressed in almost all epithelial cells, however generally at lower levels than in professional immune cells [8]. TLR signalling induces multiple pathways to activate various inflammation-relevant transcription factors. These may include the nuclear factor-κ B family of factors (NF-κB), members of the interferon regulatory factor family (e.g. IRF3/7), and activator protein 1 (AP1) [10]. These factors regulate the expression of various cytokines, chemokines, interferons, and anti-microbial molecules. PAMPs contacting the outside the host cell are recognised by those transmembrane TLRs reaching into the exterior. These are TLR4 and TLR5 homodimers and also TLR2/1 and TLR2/6 heterodimers. Ligands for TLR2 heterodimers are bacterial lipoproteins whereby TLR2/1 detects triacetylated lipoproteins typical for Gram-negative bacteria while the diacetylated lipoproteins from Gram-positive ones are ligands for TLR2/6. Lipopolysaccharide (LPS) is a component of the outer membrane of most Gram-negative bacteria and is the typical ligand for TLR4. TLR5 senses flagellin. All these ligands are components of the surface of bacteria.

Several TLRs are restricted to endosomes. They are only activated if their ligands are delivered via endocytic/phagocytic pathways. TLR3, TLR7, TLR8, TLR9, and TLR13 recognise nucleic acids. The ligands of TLR11, a receptor which is expressed in various epithelial cells, are flagellin from *Salmonella* or *Escherichia coli* and profilin from *Toxoplasma gondii* [11]. Spatial restriction of flagellin recognition by this receptor to the endosome is discussed as tolerance against commensal flagellin. Efficient signalling is only elicited by invasive *Salmonella* or *E. coli*. Efficient recognition of profilin by TLR11 requires TLR12 as cofactor. Note that TLRs 11, 12, and 3 are not expressed in human. Nucleic acids recognising TLRs can sense viral double-stranded RNA (dsRNA; TLR3) or single-stranded RNAs (ssRNA; TLR7, TLR8) and bacterial and viral DNA featuring high amounts of unmethylated CpG motifs (TLR9). TLR3, TLR7, and TLR8 recognise genomes of viruses entering the epithelial cell via the endocytic route. Relevant pathogens are influenza A (dsRNA, TLR3) [12], respiratory syncytial virus (ssRNA, TLR7) [13], or rotavirus (dsRNA, TLR3) [14]. In most cases, the epithelial cell alone is unable to eliminate those pathogens. However, eliciting an adequate virus-specific innate immune response in the epithelial cells is crucial for eradication of the pathogens and for the development of immunity by professional immune cells [15]. Interestingly, high expression levels of TLR3 in intestinal epithelial cells correlates with resistance against rotavirus infection [14]. This example emphasises the importance of TLR3 signalling in those cells. TLR9 can sense DNA of bacteria after their intrusion into epithelial cells. *Salmonella typhimurium* is a well-studied example hereof. It was shown that TLR9-deficiency leads to enhanced susceptibility to infection with this pathogen [16]. These authors also showed that a TLR9 response in intestinal epithelial cells may protect intestinal integrity.

C-type lectin receptors (CLRs) are plasma membrane-bound PRRs detecting carbohydrates but also many non-carbohydrate ligands. CLRs are predominantly expressed on myeloid cells. However, Dectin-1 was found in almost all mucosal epithelial cells. This CLR recognises β-1,3-glucans and is of particular relevance to counteracting against fungal infections. Dectin-1 signalling triggers production of inflammatory cytokines but initiates also phagocytosis. It mediates anti-fungal immunity against *Candida albicans*, *Aspergillus fumigatus*, *Pneumocystis carinii*, and *Cryptococcus neoformans* [17]. Dectin-1 is also involved in sensing mycobiota and is therefore important for maintaining gastrointestinal homeostasis. Deficiency of this receptor leads to fungal-mediated worsening of gut inflammation [18]. In this context, the induction of innate immune memory may be of particular relevance because β-glucans are well known to initiate trained immunity. However, these processes have so far predominantly been studied in monocytes and macrophages [19] rather than in epithelial cells.

The diverse group of NOD-like receptors (NLR) is intracellular PRRs. From among them, NOD1 and NOD2 receptors are expressed in various epithelial cells. Their ligands are γ-D-glutamyl-meso-diaminopimelic acid and muramyl dipeptide respectively. Both are substructures of peptidoglycan, a macromolecule forming the cell wall of Gram-positive and Gram-negative bacteria [20]. NOD signalling is involved in the production of pro-inflammatory cytokines and anti-microbial molecules in response to bacterial pathogen contact. Peptidoglycan fragments can reach the cytoplasm of the epithelial cells via multiple routes. Transmembrane peptide transporters in the host cell membrane (e.g. PEPT1) and endosomes (e.g. SLC15A3 and SLC15A4) may be relevant for PAMP internalisation. Several invasive bacteria are known to be recognised via NODs in epithelial cells. Examples are enteroinvasive *E. coli* [21], *Shigella flexneri* [22], and *Streptococcus pneumoniae* [23]. NOD activation is apparently linked to xenophagy-mediated clearance of intracellular bacteria (see below).

The NLR family contains several factors necessary for inflammasome assembly. These multiprotein complexes are formed in response of NLRs binding to a variety of PAMPs and DAMPs. While NLRs are the sensors, caspase 1 is the enzymatic component to proteolytically process precursors of several cytokines, such as IL1β or IL18, to establish their mature and active form. Caspase 1 and almost all sensor factors, e.g. NLRP1, NLRP3, NLRP6, NLRP12, and NLRC4, are expressed in epithelial cells [24]. Much is known about their immune stimulatory role in intestinal epithelial cells [25]. NLR deficiencies are linked to enhanced susceptibility against colitis (NLRP3), to alteration of faecal microbiota (NLRP6, NLRP12) [26], or to compromised elimination of invaded *S. typhimurium* by failed activation of pyroptosis and extrusion of infected intestinal epithelial cells (NLRC4).

Viral RNAs are recognised in the cytoplasm by the family of RIG-I-like receptors (RLRs). The three members of this family—RIG-1, melanoma-differentiated gene 5 (MDA5), and DExH-box polypeptide 58 (DHX58; also known as LGP2)—are all known to be expressed in epithelial cells [10]. These receptors are involved in mounting an innate immune response in the epithelial cells against various RNA viruses, e.g. rotavirus, influence A virus, rhinovirus, and norovirus. The innate response includes the expression of pro-inflammatory cytokines, type I interferons (IFNs), and IFN-stimulated genes (ISGs). Many ISGs are involved in limiting viral replication via degradation of viral RNAs and initiation of apoptosis within the infected cell [27].

### Hyporesponsiveness/tolerance: coping of epithelial cells with a wealth of PAMPs and DAMPs

Mucosal epithelial cells are frequently confronted with—mostly—harmless bacteria and their components including many PAMPs. Under homeostatic conditions, it is not beneficial to blithely sense all these patterns and initiate an inflammatory reaction. This would entail the risk of triggering serious immunopathological events and might even provoke autoimmunity. Several mechanisms evolved to eventually confine and dampen PRR signalling. Some of these are particularly relevant in epithelial cells.

#### Spatial control of PRR expression

Tightly controlling the spatial localisation and activation of surface TLRs helps preventing their excessive and unwanted signalling. One elegant solution to this problem is the compartmentation of the receptors. This principle provides the option to detect molecules outside their spatial context just—and only—at that moment when they become a problem for the organism. The apicobasal polarity of epithelial cells almost predestines them to apply this mechanism. Epithelial cells often restrict or prefer the expression of membrane-bound TLRs to their basolateral side [8, 28]. This assures that microorganisms only get access to the PRRs after overcoming the physical epithelial barrier. Hence, only potentially virulent pathogens are perceived. Under homeostatic conditions, most of the TLR2, TLR4, and TLR5 receptors are localised to the basolateral plasma membrane in simple and pseudostratified epithelia, e.g. intestine and airway or to the basal cell layers in stratified epithelia like in the oral cavity [8, 28, 29]. Only after disruption of the epithelial barrier, for instance by the gastrointestinal pathogens enteropathogenic and enterohemorrhagic *E. coli* or the respiratory pathogen *Klebsiella pneumoniae*, are the basolateral TLRs being activated and will be inducing strong inflammation. This will ideally eradicate the pathogens. During inflammation, epithelial cells may enhance the expression of TLR2 and TLR4. Their intracellular localisation may change during infection. In the airway, a considerable amount of TLR4 factors was found in the Golgi complex and was transferred to the surface subsequent to pathogen contact [8]. In the inflamed bovine mammary gland, abundant amounts of TLR2 receptors were found on the apical side of mammary epithelial cells, while in the healthy gland, only low amounts of TLR2 were seen inside those cells [30]. Apical enrichment of TLR4 during chronic inflammation was reported from ileum and colon [28].

The endosomal TLRs (TLR3, TLR7, TLR8, and TLR9) perceive their nucleic acid ligands from viruses and bacteria if these have entered the cell through endocytic or phagocytic pathways [31]. In addition, only after pathogens or PAMPs entered the cytoplasm are they exposed to their cognate cytoplasmic receptors like NODs or RIG-I [32]. DAMPs residing in the vesicular lumen, e.g. glycans, become recognisable by cytosolic galectins only after pathogen-mediated breakdown of those phagocytic vesicles [33]. The cell may interpret this as danger signal indicating intruding pathogens. Hence, autophagy of the respective cellular area is triggered, thereby disarming the pathogens localised there.

#### Confined availability of bystander factors for TLR signalling

Limited or even lacking expression of PRR cofactors is another means for reducing sensitivity of the sentinel system and increasing the thresholds necessary for induction of strong inflammations [34]. This mechanism is postulated for dampening the TLR2 and TLR4 signalling through limiting interaction of the receptors with their cofactors CD36, MD2, and CD14 in airway epithelial cells [29]. The latter factors regulate the function of the TLRs by association with their extracellular domain. Larger amounts of soluble forms of these accessory factors may be derived from other sources like resident macrophages. Hence, these leucocytes may thereby tune the inflammatory response of their neighbouring epithelial cells. Adjustment of the inflammatory reaction may also be caused by altered expression of negative regulators of PRR signalling pathways [35]. More than 200 proteins are known to attenuate inflammatory PRR signalling and their cell type-dependent regulation is presumed [36]. Most often, intracellular inhibitors must be degraded in response to an external signal to achieve fully powered signal transduction from the receptor.

#### Induction of innate immune memory

It is long known that professional immune cells (e.g. macrophages) become insensitive against repeated challenges with some abundant TLR ligands, such as LPS. The phenomenon is long known as endotoxin tolerance or tolerance to pyrogens [37]. Through the years, this phenomenon was understood as part of the innate immune memory [38]. Recent studies indicate that also epithelia cells of the airway and the mammary gland can be reprogrammed establishing an innate immune memory [39, 40]. Such reprogramming has two different aspects: on the one side, it enhances immunological fitness characterised, for example, by increased expression of anti-microbial factors in response to a second inflammatory stimulus. On the other side, it establishes endotoxin tolerance preventing overshooting cytokine and chemokine synthesis. This might eventually allow microbiota to colonise mucosal tissues. Most interestingly, these mechanisms are apparently not only operating in mature cell populations but also in progenitor and stem cells. These cells, rather than the short-lived circulating mature monocytes (half-life 1–3 days), are conceivably responsible for the in vivo observed long-term effects of the monocyte memory. In this regard, airway epithelial cells are a highly probable target cell population for innate memory because their average half-life is around 6 months [41] and their progenitors are located directly on the site of the potential stimulus. Furthermore, they are by far the most abundant cells of the respiratory tract covering a surface of more than 90 m^2^ in humans while one can only find less than one macrophage per alveolus [42]*.*

## Relevance of phagocytic mechanism in epithelial cells: eating for health

Phagocytosis is an important cellular mechanism in homoeostasis and disease. The main task of phagocytosis during infection diseases is to destroy the invaded pathogen. This includes recruiting and activating immune cells for mounting an effective immune defence and to remove disease-causing microorganisms from the site of infection. The non-immunogenic role of phagocytosis is the removal of dead cells. Cell removal is mandatory for organ- and body-shaping during embryonic development. In adults, dead cell clearance is crucial to maintain tissue homeostasis and integrity during normal tissue turnover and after injury. Professional phagocytes are considered as the most relevant and best characterised cell type taking over these various tasks. However, also epithelial cells are capable of phagocytosis and are considered as facultative or non-professional phagocytes.

### Dead cell clearance: a challenge for epithelial cells

Clearance of epithelial tissue from dying and dead cells occurs constantly in tissues with a high turnover rate of cells like in the intestine or during reorganisation of the tissue after injury. It occurs in large scale during involution of the mammary gland during the lactation cycle. Defects in efficient and quick removal of dying cells from the epithelium compromises epithelial integrity and can lead to secondary necrosis resulting in release of inflammatory DAMPs. This may eventually cause very severe diseases like chronic inflammatory disorders, autoimmunity, or cancer. Clearance can be achieved either by extrusion or by efferocytosis.

Extrusion is the shedding of apoptotic epithelial cells from the cell layer without compromising the barrier. The dying cell is surrounded by an actomyosin ring formed by the neighbouring cells. This ring may constrict and thereby squeeze the targeted cell out from the cell layer [43, 44]. Epithelia of vertebrates predominantly extrude cells apically into the surrounding lumen. This mechanism may be driven by RhoA GTPase [44]. Little is currently known about how injured cells are being detected by their neighbours. Crucially involved may be sphingosine-1-phosphate (S1P). This factor is released by apoptotic cells and may be detected by the neighbouring cells through the ubiquitously expressed S1P receptors [44]. Subsequently, neighbouring cells form new tight junctions between them and close the gap.

Efferocytosis is the second elimination mechanism of apoptotic corpses. In this process, dying cells are engulfed by professional and non-professional phagocytes. This leads to an immediate removal of the apoptotic cell prior to disruption of membrane integrity and to the release of inflammatory DAMPs [45]. The advantage of efferocytosis over extrusion resides in the opportunity that, after ingestion of the target cell, some of its components may be reutilised. Efferocytosis is primarily mediated by professional phagocytes notably macrophages and other myeloid cells. They are highly competent to detect, incorporate, and degrade apoptotic cells. Neighbouring epithelial cells may act as non-professional phagocytes in such epithelia featuring high turnover rates or harbouring only few macrophages. Although the phagocytic activity of epithelial cells is less pronounced than that of their professional counterparts, their sheer abundance makes them very important efferocytes in those tissues. The relevance of epithelial cell efferocytosis was identified so far in airway, gut, mammary gland, liver, kidney, and retinal pigment epithelium [46, 47].

Apoptotic cells may express a plethora of “eat me” signals on their surface. The most widely studied and most common surface marker is phosphatidylserine (PS). PS is normally located on the inner leaflet of the plasma membrane [47, 48]. Externalisation of PS by translocation to the outer leaflet is a very rapid process during apoptosis. It therefore constitutes a critical efferocytosis signal across diverse cell types. Little is known about how epithelial cells recognise their apoptotic neighbour. PS-sensing receptors may play an important role in this process [47]. Similarly, poorly understood are the mechanisms how apoptotic cells are taken up by non-professional phagocytes. It is still debated if this process is comparable with macropinocytosis or phagocytosis [49]. However, cytoskeletal rearrangements mediated through Rho family GTPases may be involved in the internalisation of corpses. It is suggested that Rac1 has a pro- while RhoA has an anti-efferocytic effect in professional phagocytes [49]. The efferocytic uptake by airway epithelial cells may also be Rac1-dependent [50]. Following ingestion into the so called “efferosome”, the latter undergoes different maturation steps and eventually the apoptotic cell will be digested during lysosomal processing. In professional phagocytes, this process is extremely rapid. It awaits experimental clarification if these processes occur similarly in professional and non-professional efferocytes, such as epithelial cells.

Interestingly, epithelial cell mediated efferocytosis may induce an anti-inflammatory environment. The response to engulfment of apoptotic cells by airway epithelial cells results in enhanced production of transforming growth factor β (TGFβ) and prostaglandin E2 [50]. Both are well-known anti-inflammatory mediators. Furthermore, Juncadella et al. showed that mice with Rac1-mediated airway defects of efferocytosis in airway epithelial cells responded with enhanced induction of pro-inflammatory IL33 and reduced production of TGFβ and IL10 in the bronchoalveolar lavage fluid after an intranasal challenge with apoptotic cells [50]. This indicates that airway epithelial cells with dysfunctional efferocytosis respond to apoptotic cells in their environment with a pro-inflammatory (IL33) instead of an anti-inflammatory response (TGFβ). Furthermore, this apparently influences myeloid or lymphoid cells in the tissue because IL10 is primarily expressed by myeloid cells and only to a lesser extent by lymphoid cells. Epithelial cells do not express this cytokine at all. This indicates that, during efferocytosis, epithelial cells not only secrete anti-inflammatory mediators but also that they trigger professional immune cells—most probably tissue resident macrophages—to also produce anti-inflammatory cytokines. This communication between professional and non-professional efferocytes may also function vice versa. Recently, Han et al. found that macrophages having previously been stimulated with the asthma-typical Th2 cytokines IL4/IL13 secrete insulin growth factor 1 (IGF1) and this depresses efferocytosis by airway epithelial cell [51]. Concurrently, IGF1 enhances in airway epithelial cells the uptake of macrophage-derived microvesicles containing anti-inflammatory mediators. Besides its induction during asthma, IGF1 is also induced in lung of mice exposed to high doses of aerosolised LPS [52]. LPS stimulation is known to be correlated with strong induction of pro-inflammatory cytokines such as TNF and IL1β. Hence, the IGF1-mediated influence on epithelial efferocytosis may occur not only during allergic but also during PAMP-mediated inflammation. Han et al. discussed the therapeutic potential of the IGF1 effect upon/on epithelial cells as potential modulator against airway hyper-responsiveness during asthma. However, considering that efferocytosis by epithelial cells is beneficial for maintaining epithelial tissue homeostasis, it appears questionable if the IGF1-mediated inhibition of this mechanism might really be feasible to counteracting allergic diseases. Efferocytosis by epithelial cells has extensively been analysed in regard to asthma. However, dysfunctional efferocytosis may also have implications in disorders of other epithelial tissues. Relevant examples include the intestine regarding inflammatory bowel disease (IBD) [53, 54] or the postpartum mammary gland where impaired death cell clearance may lead to fibrosis or epithelial cell hyperplasia [55].

### Pathogen-induced phagocytosis: chewing up the enemy

**Fig. 1:**
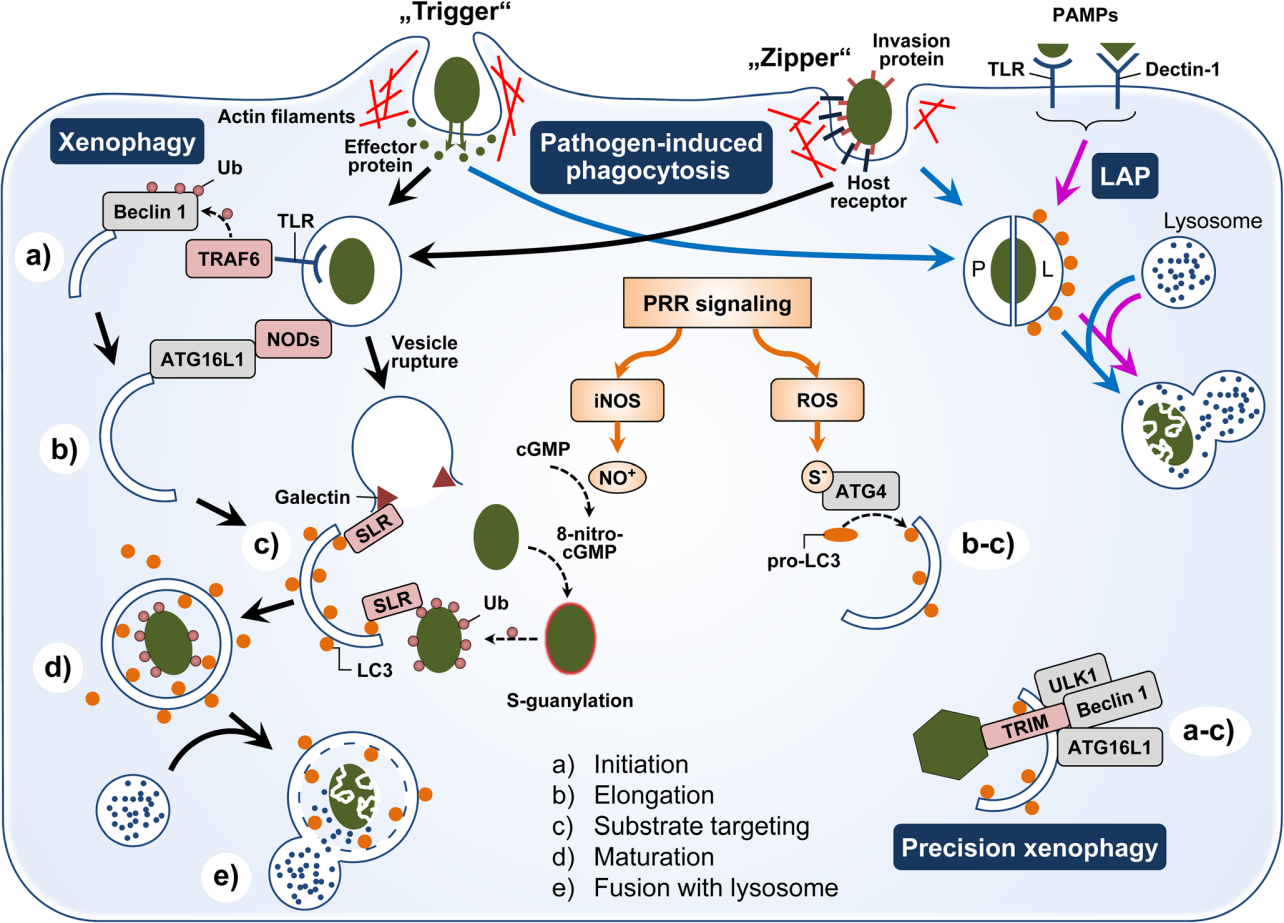
Overview of pathogen-induced phagocytosis and xenophagy mechanisms in epithelial cells. Non-professional phagocytes like epithelial cells can internalise pathogens (dark green) via “trigger” or “zipper” mechanisms. Pathogens using the “trigger” mechanism secrete effector proteins in the host cell. These factors modulate the actin cytoskeleton leading to the generation of membrane ruffles and internalisation. The “zipper” mechanism based on the interaction of host receptors on the plasma membrane with invasion proteins expressed on the pathogen surface. These interactions lead to localised cytoskeleton rearrangement and pathogen uptake. The internalised pathogen-containing vesicles may follow as classical phagosome (P) the lysosomal degradation route (blue arrows). Pathogen-mediated activation of PRRs (surface TLRs, Dectin-1) can lead to LC3-associated phagocytosis (LAP, magenta arrows) and the formation of a LAPosome (L) which is characterised by LC3 (orange spot) on the outer leaflet of the vesicle membrane and a more rapid fusion with the lysosome. In addition, xenophagy (black, solid arrows) may be activated by PRR pathways. TLR signalling activates the E3 ubiquitin ligase TRAF6 that ubiquitinates (Ub) Beclin 1 necessary for xenophagy initiation (a). Activated NODs interact with ATG16L1 which is relevant for phagophore elongation (b). If the pathogen escapes into the cytosol, rupture of the vesicles is sensed via xenophagy receptors (SLRs) that bind galectins. These in turn recognise the cytosolic presence of glycans being normally hidden inside the vesicles. Pathogens entering the cytosol are ubiquitinated (Ub) by different host factors. Some SLRs can bind that ubiquitin coat surrounding the pathogen. Subsequently, SLRs bind LC3 on the elongating phagophore and thereby tag the pathogens and/or cellular regions harbouring the bugs for xenophagic degradation. PRR signalling (orange arrows) often leads to high cellular levels of nitric oxide (NO^+^) and reactive oxygen species (ROS). ROS upregulate ATG4 expression concurrently mediating oxidation of ATG4 at cysteine (S^−^). Both events facilitate LC3 enrichment on the phagophore membranes promoting its elongation as well as substrate targeting. NO^+^ formed by the activity of inducible nitric oxide synthases (iNOS) can nitrify cGMP to 8-nitro-cGMP that modifies cysteines on the bacterial surface (S-guanylation). This leads to enhanced ubiquitination, thereby tagging the pathogen for recognition by SLRs. Members of the TRIM family of auto-/xenophagy receptors are involved in precision xenophagy. TRIMs recognise pathogenic targets (like viral capsids, dark green hexagon) and form a platform for core xenophagy factors (ULK1, Beclin 1, and ATG16L1). Thereby, they bundle initiation, elongation, and substrate targeting to one specific cellular area. After enclosure, the xenophagic vesicle undergoes a maturation process marked by the dissociation of LC3 from the outer membrane (d) and eventually fuses with the lysosome (e) leading to the degradation of the pathogens

The epithelium of mucosal tissues is the preferential entrance site for various pathogenic microorganisms. Phagocytosis is a central mechanism in host defence against invading pathogens. It involves recognition, uptake, and destruction of microbes. This process is highly efficient in professional phagocytes such as macrophages. Non-professional phagocytes like epithelial cells are also capable of phagocytosis but they use different mechanisms. Professional phagocytes rely on opsonisation of the target (e.g. pathogen) as fundamental principle behind their high phagocytic capacity and this mediates also their wide and diverse recognition repertoire. Opsonic phagocytosis is not induced directly by pathogen recognition but through sensing endogenous host proteins tethered to microbes. This mechanism depends on two classes of receptors, the Fcγ receptors (FcγR) and the complement receptors (CRs), binding to the Fc portion of IgG or cleavage products of the complement component C3, respectively. Non-professional phagocytes do not express those receptors necessary for opsonic phagocytosis of pathogens. Rather, the pathogen itself triggers its entry into these cells. However, the host cell plays a very active role in the internalisation process. Pathogen-induced phagocytosis in non-professional phagocytes is mediated by modulating the actin cytoskeleton of the host cell to using the so-called trigger or zipper mechanisms (Fig. 1 [56]). Bacteria using the trigger mechanisms inject effectors into the host cell to induce cytoskeleton reorganisation at the site of pathogen contact. This results in the formation of membrane ruffles. These ruffles enfold the pathogen, fuse, and eventually form a pathogen containing vesicle. Central regulators of this cytoskeleton rearrangement are host-expressed Rho GTPases. Examples of bacteria using this trigger mechanism are *Salmonella* sp. and *Shigella* sp. colonising intestinal epithelial cells. In contrast, the zipper mechanism exploits host surface proteins being involved in cell adhesion like integrins and cadherins to attach to the host membrane. This principle is used for internalisation by a wide range of bacteria, for instance *Listeria monocytogenes*, *Staphylococcus aureus*, *Helicobacter pylori*, and *Yersinia enterocolitica* [57]. The interaction of bacterial surface adhesins with these host receptors initiates spatially restricted actin and/or microtubule rearrangements at the contact site resulting in ingestion of the bacteria. Also, some viruses, like influenza A and rota virus [58, 59], use the zipper mechanism for host cell entry. In addition, airway epithelial cells internalise *Aspergillus fumigatus* conidia after sensing them via their Dectin-1 receptor [60]. This receptor interaction is necessary for phagocytosis of the fungal conidia, also mediated by actin/microtubule polymerisation. The actin cytoskeleton-dependent deformation of host plasma membrane is often suggested to be the crucial mechanism during pathogen-induced phagocytosis. However, *Pseudomonas aeruginosa* uses a lipid zipper to enter epithelial cells independent of actin polymerisation. This pathogen expresses a surface lectin LecA that binds to the host glycosphingolipid Gb3 and thereby initiates a zipper to induce plasma membrane invagination [61].

Subsequent to the engulfment of the microbe, the phagosome matures by fusion and fission of endocytic vesicles [62]. This is accompanied by acidification of the lumen mediated by early recruitment of vacuolar ATPases and eventually leads to fusion with lysosomes and phagolysosome formation. This organelle contains a range of hydrolytic enzymes requiring low pH. These enzymes are responsible for degradation of foreign particles. The basic principles of phagosomal maturation in professional and non-professional phagocytes, including epithelial cells, appear to be relatively similar. Both types of phagocytes encounter a drop of the phagosomal pH value after pathogen/particle internalisation as well as phagosome/lysosome fusion [63]. However, kinetics of internalisation, phagosomal acidification, and lysosome fusion differ between professional and non-professional phagocytes. In particular, the process of phagolysosomal formation is slower in epithelial cells than in professional phagocytes. Furthermore, the sheer amount of lysosomes is much higher in professional than in non-professional phagocytes [64]. This together underscores that phagocytic killing is less efficient in epithelial cells than in macrophages or neutrophils, for example.

Some intracellular pathogens can survive in epithelial cells while being eradicated in myeloid cells. This is probably due to the slower phagolysosome formation in epithelial cells. Most often, intracellular pathogens need some time to adapt to the intracellular environment and to induce expression of virulence factors necessary for survival. Lysosomal degradation liberates PAMPs from the pathogens. These may be recognised by PRRs and thus initiate the mounting of adequate innate immune defence mechanisms.

The importance of pathogen digestion for mounting an adequate immune defence against Gram-positive pathogens was recently exemplified in mammary epithelial cells (MEC). It is long known that infection of the udder with such pathogens (*S. aureus*, *Streptococcus uberis*) will often cause only a mild inflammation, known as subclinical mastitis [65]. The reason resides in the failure of MEC to recognise intact *S. aureus* or *S. uberis* pathogens [66, 67], albeit that *S. aureus* is readily invading the MEC [68]. However, the MEC efficiently sense and react against isolated PAMPs of those Gram-positive pathogens. Inadequate lysosomal degradation of intracellular *S. aureus* by the MEC was indicated by the fact that mechanically disrupted *S. aureus* would trigger a substantial immune reaction in the MEC [66]. In stark contrast, macrophages induce a strong innate immune response against both pathogens [5].

For all these reasons, epithelial cells are often exploited as an infectious “foothold” by a wide range of pathogens. For instance, highly virulent *S. aureus* strains are able to intracellularly persist in airway epithelial cells (A549) but were cleared within 3 days in macrophages [69]. *Campylobacter jejuni* can survive in intestinal epithelial cells by avoiding its delivery into lysosomes. However, this pathogen is rapidly killed by macrophages [70]. Also, for *Salmonella enterica* serovar *Typhimurium*, it is harder to replicate and survive in macrophages than in intestinal epithelial cells [71]. Nevertheless, highly virulent intracellular pathogens are known to express a plethora of virulence factors enabling their survival also in macrophages. It should be kept in mind, however, that epithelial cells are able to kill a range of pathogens. However, these microbes are usually of only marginal interest to the scientific community and only very few publications deal with them because they elicit only unproblematic, self-curing infection. In contrast, a stronger focus lies obviously on very highly virulent pathogens. An example of effective pathogen killing by epithelial cells is the eradication of the opportunistic pathogen *Pseudomonas aeruginosa* [72]. This bacterium adheres to apoptotic epithelial cells and is internalised via efferocytosis by neighbouring epithelial cells together with dead cell compartments. Subsequently, the pathogen is rapidly eliminated by lysosomal mechanisms. Also, the majority of internalised *A. fumigatus* conidia are effectively killed via the lysosomal route in airway epithelial cells [60].

### Xenophagy: remedy if pathogens escape the phagocytic degradation route

Xenophagy is a type of selective autophagy [73] and constitutes a phagocytosis-related defence mechanism against invading pathogens. It targets intracellular pathogens for lysosomal degradation if they escape from the phagosome. The mechanism depends on the formation of double-membraned endomembrane vesicles. It involves the steps of initiation, elongation, substrate targeting, maturation, and lysosomal fusion (Fig. 1). The different stages of xenophagy are identical to the canonical macroautophagy pathway. Initiation occurs at the endoplasmatic reticulum, the Golgi apparatus, or endosomal organelles which are the sources for the phagosome membrane. The starting point of phagophore formation is the translocation of the unc-51-like autophagy-activating kinase (ULK) protein complex and subsequent recruitment of the autophagosome-specific phosphatidylinositol 3 (PI3)-kinase complex and induced PI3-phosphate synthesis. Elongation of the phagophore depends on the ubiquitin-like conjugation systems which eventually facilitate anchoring of the microtubule-associated protein light chain 3 (LC3) to the autophagosome membrane. LC3 is necessary for substrate targeting by interaction with autophagy receptors via their LC3-interacting regions (LIRs). Sequestosome 1-like receptors (SLRs) represent a subgroup of these receptors. Sequestosome-1 (also known as ubiquitin-binding protein p62), optineurin, NDP52 (also called CALCOCO2), and NBR1, autophagy cargo receptor, are members of this subgroup [74]. They recognise specific tags on the surface of invading microorganisms or damaged phagosomal membranes and thereby direct the respective cellular localisation along with the pathogen to xenophagic degradation. These tags include the ubiquitin coat surrounding cytosol-invading bacteria and cytosolic galectins binding to glycans which are normally hidden inside the vesicles and become accessible after vesicle rupture. Ubiquitination of bacteria is accomplished by E3 ligases like the leucine-rich repeat and sterile alpha motif containing 1 (LRSAM1) or parkin RBR E3 ubiquitin protein ligase (PRKN). Another class of autophagy receptors is the tripartite motif (TRIM) family of proteins [75]. TRIMs can recognise their targets without the need for ubiquitin. Well known is TRIM5α that binds to retroviral capsids. In addition, TRIMs can also function as a platform for core regulators of the autophagosome machinery (ULK1, Beclin 1, and ATG16L1). Hence, they are more complex regulators of autophagy than the SLRs [76]. Therefore, this highly selective type of autophagy is termed “precision auto/xenophagy” (Fig. 1). Subsequently, to target the cargo for destruction, the xenophagosome is sealed, matures, and fuses eventually with the lysosome (Fig. 1).

A range of danger signals are known as triggers for xenophagy. Pathogen sensing by TLRs initiates phagophore formation via TNF receptor-associated factor 6 (TRAF6)-mediated ubiquitination of Beclin 1 (Fig. 1). Then, Beclin 1 dissociates from the negative regulator B cell lymphoma 2 protein and triggers the formation of the autophagosome-specific PI3-kinase complex. Activated NOD receptors interact with ATG16L which is part of the ubiquitin-like conjugation system relevant for phagophore elongation. The importance of this interaction was shown for Crohn’s disease [77]. Mutated NOD2 was unable to recruit ATG16L to the plasma membrane at the site of bacterial invasion. This leads to impaired xenophagosome formation and inefficient pathogen elimination. In addition, NOD-like receptor (NLR) NLRP6 may be crucial for autophagy in intestinal epithelial cells. NLRP6 deficiency in mice leads to impaired autophagosome formation in those cells and a higher susceptibility to persistent *Citrobacter rodentium* infection [78]. In contrast, other NLRs like NLRP4 and NLRC4 may inhibit autophagy via interaction with Beclin 1 [79]. Inflammation caused by invading pathogen is normally associated with high levels of nitric oxide (NO^+^) and reactive oxygen species (ROS) in the cell. ROS upregulates ATG4 expression. This factor is necessary for proteolytic cleavage of pro-LC3 which is the first step to generate a membrane-bound form of LC3 by conjugation to phosphatidylethanolamine. Besides, ATG4 is also involved in delipidation of LC3 and thereby negatively impacting autophagy. Oxidation of ATG4 by ROS inhibits the delipidating activity without affecting the initial processing of pro-LC3. Consequently, autophagy is enhanced [80]. NO^+^ induces cGMP nitration to generate the endogenous xenophagy enhancer 8-nitro-cGMP [81]. This molecule modifies cysteine residues of proteins (S-guanylation) on the surface of cytosolic bacteria. S-guanylation may represent a tag for polyubiquitination. These ubiquitin chains define targets for SLRs and phagophore sequestration.

Intracellular pathogens evolved a range of strategies to avoid or subvert xenophagy by the host cell. This includes blocking of initiation and formation of the xenophagosome, shielding to prevent the recognition by autophagy factors as well as prevention of LC3 targeting, blocking of xenophagosome maturation, and fusion with the lysosome (reviewed in [82]). Respective pathogens are *Burkholderia pseudomallei* that downregulates in airway epithelial cells the autophagy gene ATG10 which involved xenophagosome elongation; *Shigella flexneri* that is able to survive in the cytosol of epithelial cells by circumvention Atg5-recognition via masking its surface by expressing the bacterial effector IcsB; *Serratia marcescens* that persist in LC3-containing vesicles of epithelial cells which are non-acidic and have no degradative properties, indicating that this pathogen blocks xenophagosome maturation and lysosome fusion. Furthermore, *S. aureus* induces autophagosomes and blocks their maturation via activation of its accessory gene regulatory (agr) system to form a niche for replication and survival.

### LC3-associated phagocytosis: a bridge between phagocytosis and xenophagy

LC3-associated phagocytosis (LAP) was only recently detected. This mechanism links autophagy and phagocytosis (reviewed in [83]; Fig. 1). It operates in professional and non-professional phagocytes including epithelial cells. LAP uses several, but not all components of the autophagy pathway to associate LC3 to phagosome membranes. The resulting single membrane vesicle is called LAPosome. Activation of PRRs such as TLRs (TLR1/2, TLR2/6, and TLR4) and Dectin-1 is involved in pathogen/particle targeting, uptake, and LAPosome formation. Interestingly, the ULK complex mandatory for autophagy initiation is dispensable for LAP. The first step of overlap between autophagy and LAP is the formation of the autophagy-specific PI3-kinase complex. Unfortunately, the exact mechanism connecting PRR signalling to PI3-kinase complex recruitment remains elusive. An interaction has been suggested involving on one side phagosomal cup formation, engulfment, early phagosome maturation, and the mechanisms of cytoskeletal rearrangements necessary for these processes, and on the other side, formation and recruitment of the autophagy-specific PI3-kinase complex. Yet, the very early events in phagosome formation appear to be independent of this PI3-kinase complex or PI3-P generation. Later on, during maturation, LC3 is conjugated to the LAPosome involving the autophagy-specific ubiquitin-like conjugation systems. In addition, ROS production is important for LC3 lipidation. As mentioned above, ROS promotes via ATG4 the lipidation of LC3. This mechanism appears to be of particular relevance for LAP. LC3 is present in the LAPosome only in the outer leaflet of the vesicle membrane. Only if positioned there, LC3 might facilitate vesicle maturation, migration along microtubules, and fusion with lysosomes. Lysosomal fusion and cargo degradation in the LAP pathway is faster than in traditional phagocytosis allowing for more efficient pathogen killing. This is evidenced, for example, by the reduced clearance of *A. fumigatus* infections in LAP-deficient mice.

Besides assisting defence against pathogens, LAP has a role in efferocytosis. In that process, other plasma membrane receptors, such as T cell immunoglobulin mucin protein 4 (TIM4) binding the “eat me” signal PS, mediate cargo sensing during LAP. The dead cell clearance by LAP is more efficient compared to classical efferocytosis. It leads to a faster anti-inflammatory cytokine release and dampening of the immune response which might be relevant to avoid autoimmunity.

## Conclusion

Epithelial cells form the interface between the body and the environment. They constitute not only a passive barrier but also are important guardians detecting dangers and initiating diverse defence responses. The relevance of epithelial cells as non-professional phagocytes represents a rather new aspect among these manifold functions. Although it is known that they have a significantly lower phagocytic activity compared to their professional counterparts, there is growing evidence that the phagocytic capacity of epithelial cells plays an important role in maintaining tissue homeostasis and for mounting the defence against invading pathogens. In the last years, several researches shed new light on the mechanisms and consequences of the diverse phagocytic events in epithelia cells. However, a lot of the knowledge is still inferred from comprehensive investigations in professional, rather than non-professional phagocytes. It remains to be seen if these processes and pathways are truly similar in both types of phagocytes. Better understanding the specific features of phagocytosis in epithelial cells might eventually open new ways in therapeutic interventions against infectious and non-infectious diseases.
